# Purification and biochemical characterization of Hel a 6, a cross-reactive pectate lyase allergen from Sunflower (*Helianthus annuus* L.) pollen

**DOI:** 10.1038/s41598-020-77247-z

**Published:** 2020-11-19

**Authors:** Nandini Ghosh, Gaurab Sircar, Claudia Asam, Martin Wolf, Michael Hauser, Sudipto Saha, Fatima Ferreira, Swati Gupta Bhattacharya

**Affiliations:** 1 Department of Microbiology, Vidyasagar University, Paschim Medinipur, India; 2 Department of Botany, Institute of Sciences, Visva-Bharati, Santiniketan, India; 3 Department of Biosciences, University of Salzburg, Salzburg, Austria; 4 Cell Therapy Institute, (SCI-TReCS), Paracelsus Medical University (PMU), Salzburg, Austria; 5 Division of Bioinformatics, Bose Institute, Kolkata, India; 6 Division of Plant Biology, Bose Institute, Kolkata, India

**Keywords:** Biochemistry, Immunology, Environmental sciences, Diseases

## Abstract

Sunflower pollen was reported to contain respiratory allergens responsible for occupational allergy and pollinosis. The present study describes the comprehensive characterization of a major sunflower allergen Hel a 6. Natural Hel a 6 was purified from sunflower pollen by anion exchange and gel filtration chromatography. Hel a 6 reacted with IgE-antibodies from 57% of 39 sunflower-sensitized patient sera suggesting it to be a major allergen. The patients were of Indian origin and suffering from pollinosis and allergic rhinitis. Hel a 6 exhibited allergenic activity by stimulating mediator release from basophils. Monomeric Hel a 6 displayed pectate lyase activity. The effect of various physicochemical parameters such as temperature, pH, and calcium ion on the functional activity of Hel a 6 revealed a stable nature of the protein. Hel a 6 was folded, and its melting curve showed reversible denaturation in which it refolded back to its native conformation from a denatured state. Hel a 6 displayed a high degree of sequence conservation with the pectate lyase allergens from related taxonomic families such as Amb a 1 (67%) and Art v 6 (57%). The IgE-cross reactivity was observed between Hel a 6 and its ragweed and mugwort homologs. The cross-reactivity was further substantiated by the mediator release when Hel a 6-sensitized effector cells were cross-stimulated with Art v 6 and Amb a 1. Several putative B cell epitopes were predicted and mapped on these 3 allergens. Two antigenic regions were found to be commonly shared by these 3 allergens, which could be crucial for cross-reactivity. In conclusion, Hel a 6 serves as a candidate molecule for diagnosis and immunotherapy for weed allergy.

## Introduction

The global prevalence of IgE-antibody mediated respiratory allergy caused by airborne pollen grains is increasing at an alarming rate. Grass pollens, weeds, and road-side ornamental trees are the primary sources of allergens. Certain economically important plants, which are not strictly wind-pollinated, may also trigger IgE-mediated occupational allergy in working individuals. A number of plants belonging to the families of Asteraceae or Compositae such as ragweed (*Ambrosia artemisiifolia*), mugwort (*Artemisia vulgaris*), and feverfew (*Parthenium hysterophorus*) are important sources of inhalant allergens in many parts of Europe and America^1,2^. Several allergens have been isolated and characterized from these plants. Many of these allergens are used worldwide for diagnostic and therapeutic purposes. One such allergen is the pectate lyase reported from ragweed (designated as Amb a 1) as well as from mugwort (Art v 6)^3^. Pectate lyases are pectin-degrading enzymes expressed in large amounts during pollen germination to degrade the pollen wall and emergence of the pollen tube^4^. In addition to the immunochemical works on these allergens, a lot of antigenic analyses were done to identify and map the potential IgE- and T cell epitopes of these 2 allergens. It was shown in previous publications that these 2 allergens share common IgE-epitopes resulting in immunological cross-reactivity within the sensitized patient population^3,5^. Advancement of recombinant DNA technology and genetic engineering has enabled the researchers to manufacture the allergy-eliciting components on a large scale to be used in the diagnostic antigen panel and also to manipulate the allergen structure to create an attenuated version to be used for immunotherapy. However, the foremost part of this task is to identify, purify, and derive the sequence information of the natural allergen from its natural source.

Another member of the Asteraceae family is sunflower (*Helianthus annuus*), which grows all over the world and is commercially cultivated for edible oil. It was shown that people working in the sunflower oil industry were reported to have IgE-sensitization to sunflower proteins, a case popularly known as occupational allergy^6^. In addition to the occupational allergy, sunflower pollen grains by virtue of delicate size are able to remain floating in the bioaerosol and can elicit allergic response via inhalation^7^. Sunflower was never considered as a globally important allergy-inducing plant; however, a certain percentage (21%) of allergy-sufferers was found to have serum IgE-antibodies against sunflower pollen proteins^8^. One of our earlier reports identified 8 novel allergens from sunflower pollen through a proteomic study and the major allergen being a pectate lyase, which displayed frequent IgE-reactivity within a population of pollinosis patients from an Indian megacity^8^. Most of these patients were reported to either work in the sunflower oil industry or to have sunflower plantations in their vicinity. In the present study, we aimed to purify and characterize the sunflower allergen Hel a 6 (MW 42 kDa; pI 4.3) to estimate its potential as a candidate molecule for diagnosis and therapy.

## Results

### Purification of natural Hel a 6

**Figure 1:**
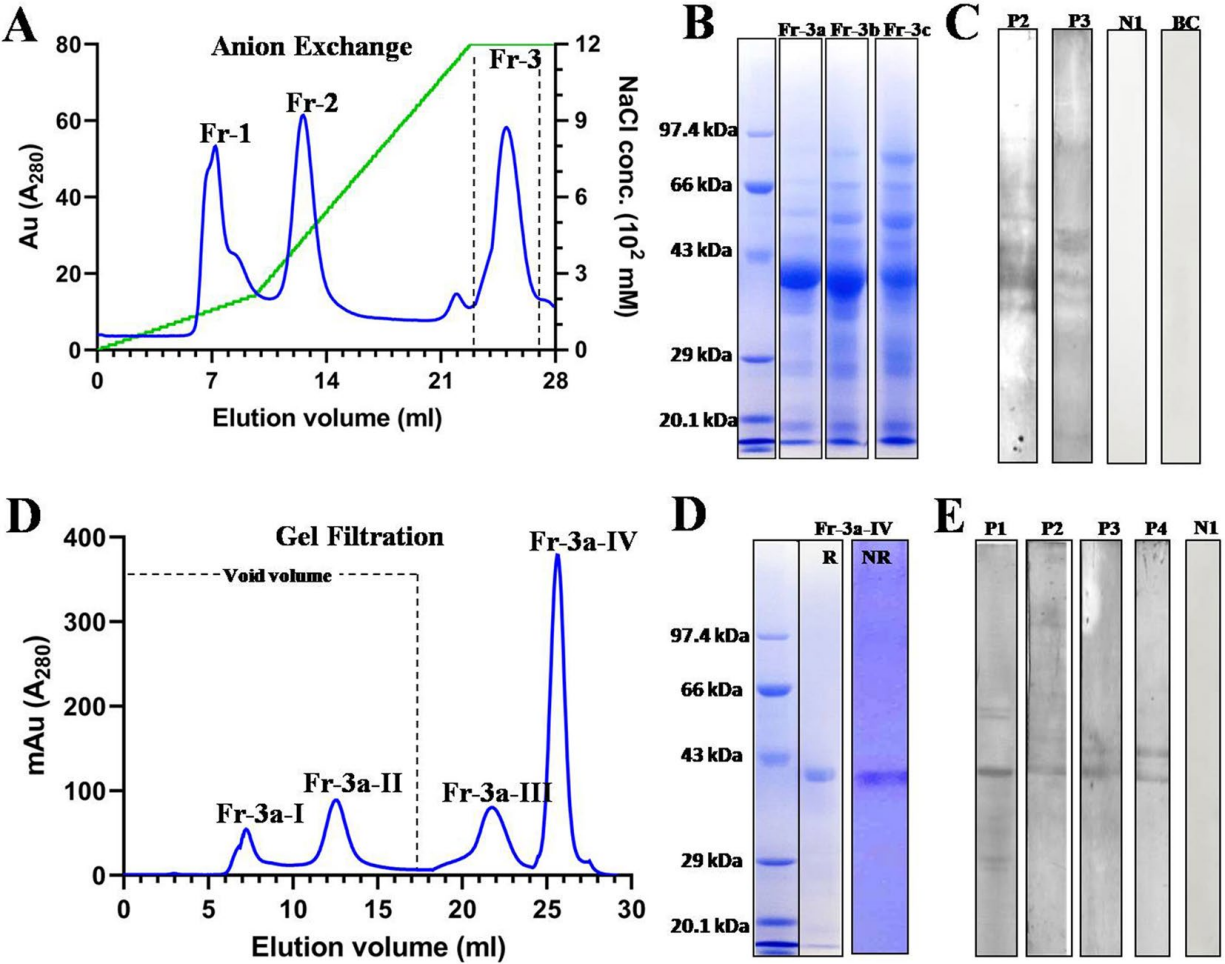
Purification of natural Hel a 6 from sunflower pollen. (**A**) Chromatogram of the NaCl eluted fractions from anion exchanger Q column showing 3 peaks (Fr-1 to Fr-3). The elution volume (ml) in *x*-axis, the A_280_ (milli absorbance unit; mAu) of the elutes in left *y*-axis and NaCl concentration gradient (mM) in right *y*-axis. (**B**) 12% SDS-PAGE profile of the 3 eluted fractions (Fr-3a to 3c) of the peak Fr-3 (flanked by dashed lines) collected as 0.5 ml/fraction tube. Fr-3a was found to have Hel a 6 with highest purity. (**C**) Therefore, Fr-3a was immunoblotted with sera from 2 sunflower-sensitized patients (P2 and P3) to confirm the presence of Hel a 6. Non-atopic serum (N1) and buffer control (BC) were used as negative controls. (**D**) Chromatogram of the eluted fractions after separating Fr-3a in gel filtration column in which A_280_ values of the eluted fractions (*y*-axis) are plotted against elution volume (ml) in *x*-axis. 17 ml of void volume is marked with dashed lines. Fr-3a-IV was found to contain Hel a 6 with > 90% purity. (**D**) Monomeric status of Hel a 6 was checked in non-reducing (NR; without β-ME) SDS-PAGE as compared to reducing (R; with β-ME). (**E**) Presence of Hel a 6 in Fr-3a-IV was checked by immunoblotting with sera from 4 sunflower-sensitized patients (P1-P4).

Using a combination of ion exchange and gel filtration chromatography, the 42 kDa allergen of sunflower pollen Hel a 6, was purified in its natural form. The total protein of sunflower pollen was extracted with 20 mM Bis–Tris (pH 4.5) and loaded on an anion exchange Q HP-column. The elution chromatogram (Fig. 1A) showed three distinct peaks (fractions 1–3). Elution was performed with a NaCl gradient from 0–100%. Out of these 3 fractions, the third peak (Fr-3) was found to contain the desired allergen on a 12% reducing SDS-PAGE (Fig. 1B). The third peak was collected in 3 fractions (Fr-3a to 3c) having 0.5 ml in each. The highest level of purity was observed in Fr-3a when screened with sera from 2 sunflower-sensitized patients (P2 and P3) by immunoblot, as shown in Fig. 1C. Therefore, Fr-3a was selected for a subsequent round of chromatographic purification. Following buffer exchange with PBS, the concentrated proteins in Fr-3a were re-purified on a gel filtration column (Fig. 1D). Several fractions were collected of which the Fr-3a-IV was found to contain the Hel a 6 at > 95% purity (Fig. 1E). No oligomerization was observed for Hel a 6 as verified by running the purified allergen on a non-reducing SDS-PAGE. The purified allergen reacted with the IgE-antibodies of sera from 4 sunflower-sensitized subjects (Fig. 1F).

### Sequence confirmation of Hel a 6 by mass spectrometry

The sequence identity of the purified Hel a 6 was verified by using two different mass spectrometric techniques such as MALDI TOF/TOF and LC-ESI qTOF. Both the MS/MS spectra were searched against the NCBInr database in the MASCOT search engine; 3 and 6 unique peptides were identified in MALDI TOF/TOF and LC-ESI MS/MS respectively. All the unique peptides displayed extensive homology (p < 0.05) with pectate lyase 2 of *Helianthus annuus* under the accession number OTF85892. The unique peptides identified by both of the mass spectrometric techniques altogether exhibited 24% sequence coverage. Details of mass spectrometric identification of the allergen were illustrated in Table 1. The allergen was given an official designation Hel a 6 by the WHO-IUIS allergen nomenclature sub-committee.

**Table 1:** Results of mass spectrometry analyses of the purified Hel a 6 allergen.

NCBI Acc. No.	Protein (organism)	Unique peptides in MALDI TOF/TOF	E value of protein	Unique peptides in LC ESI MS/MS	E value of peptide	Sequence coverage
GI: 1191633749	Pectate lyase 2 (*Helianthus annuus*)	R.FGFFQVVNNNYDR.W	0.00094	R.FLAPDDAAK.K	0.013	24%
		R.ADAPESESMTWNWR.T	0.26	R.QAMADCAQGFAK.G	6.9e−05	
		R.WGTYAIGGSSAPTILSQGNR.F	6e-007	K.QIWIDHCSFSK.A	0.0012	
				K.VEITNGGLTLMDVK.N	1.3e−06	
				K.VMLLGADDGHHQDK.N	1.2e−05	
				R.WGTYAIGGSSAPTILSQGNR.F	0.00022	

Search parameters are 01 missed cleavage, p < 0.05 as significance threshold, 0.5 Da (precursor ions), and 1.2 Da (fragment ions) mass tolerance for MALDI TOF/TOF whereas 40 ppm (precursor ions) and 100 ppm (fragment ions) respectively for LC-ESI qTOF, cysteine carbamidomethylation as fixed modification, methionine oxidation as variable modification, + 1 peptide charge for MALDI TOF/TOF and + 2, + 3, + 4 for LC-ESI qTOF.

### Hel a 6 is a major allergen of sunflower

**Figure 2:**
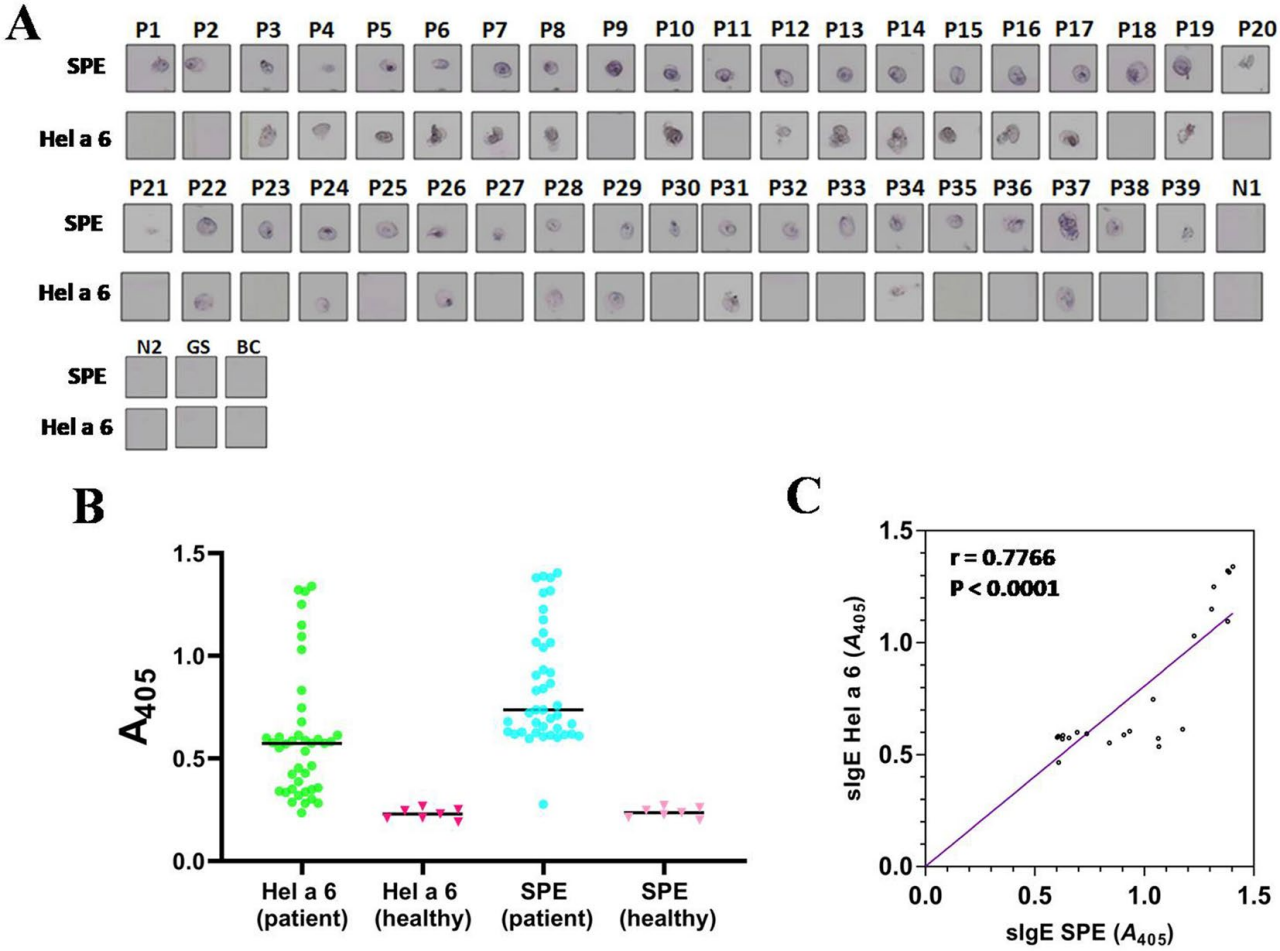
Hel a 6 is a major sunflower pollen allergen. (**A**) Immuno-dot blot in which either 0.5 µg of purified Hel a 6 or 5 µg of sunflower pollen extract (SPE) was exposed to sera from 39 sunflower-sensitized patients (P1–P39). Negative controls are 2 non-atopic sera (N1–N2), one grass pollen sensitized serum (GS), and buffer control (BC) without serum. (**B**) Scattered dot plot of IgE-ELISA (in shades of green) showing quantitative estimation of specific IgE level (Absorbance at 405 nm in *y*-axis) against either Hel a 6 or SPE (*x*-axis) in sunflower-sensitized patients (n = 39). Horizontal lines represent mean and error bars as SD. For any patient, the IgE-titre ≥ twofold of the corresponding cut-off level was considered as positive for that antigen. 22 of 39 sunflower-allergic patients were found seropositive to Hel a 6 in ELISA as well as in immuno-dot blot. Two other scattered plots (in shades of red) showing distribution of IgE-titre values in 6 non-atopic healthy sera against either Hel a 6 or SPE for negative controls. (**C**) Association between the specific IgE titres (sIgE as A_405_) of these 22 patients against SPE (*x*-axis) and Hel a 6 (*y*-axis) by non-parametric Spearman correlation analysis showing positive correlation.

The prevalence of Hel a 6 sensitization among the sunflower pollen-sensitized patients (n = 39) (see patient details in Supplementary Table S1) was evaluated by qualitative and quantitative IgE-serology. In non-denaturing IgE-immuno dot blot, 22 out of 39 patients (i.e., 57%) displayed IgE-reactivity suggesting Hel a 6 as a major sunflower allergen (Fig. 2A). No non-specific IgE-binding was observed in negative control reactions. For confirmation of immuno-dot blot results, allergen-specific ELISA was performed to quantitatively estimate the Hel a 6-specific IgE-level in each of the 39 sunflower-sensitized patient sera (P1–P39). For positive control, IgE-titers in these 39 sera were measured against the plate-bound crude antigenic extract of sunflower pollen. The cut-off or baseline for positive IgE-binding was set as the mean absorbance (A_405_) of reactions using 6 non-atopic sera (N1–N6). For an individual patient, a positive reaction was considered when the allergen-specific IgE-level in that patient serum against either Hel a 6 or crude pollen extract was ≥ twofold than that of the non-atopic sera (N) against the corresponding allergen. While all the 39 sera were found to be positive (P/N ≥ 2) for sunflower pollen extract, 22 of them reacted positively against Hel a 6 (Fig. 2B). This observation was in agreement with the results of immuno-dot blot. Since the prevalence of Hel a 6 in sunflower-allergic population was > 50%; hence this novel allergen was considered as a major allergen of sunflower. As expected, the IgE-levels against crude extract were significantly higher than that of Hel a 6 (p < 0.001) as determined by Mann–Whitney U test. However, there was a strong positive correlation (r = 0.77; p < 0.0001) between the specific IgE-levels for crude allergen and Hel a 6 in these 22 patient sera as estimated by Spearman’s correlation analysis (Fig. 2C). This observation suggests that the in-vitro IgE-binding capacity of purified Hel a 6 as strong as the crude pollen extract of sunflower.

### Hel a 6 shows allergenic activity

**Figure 3:**
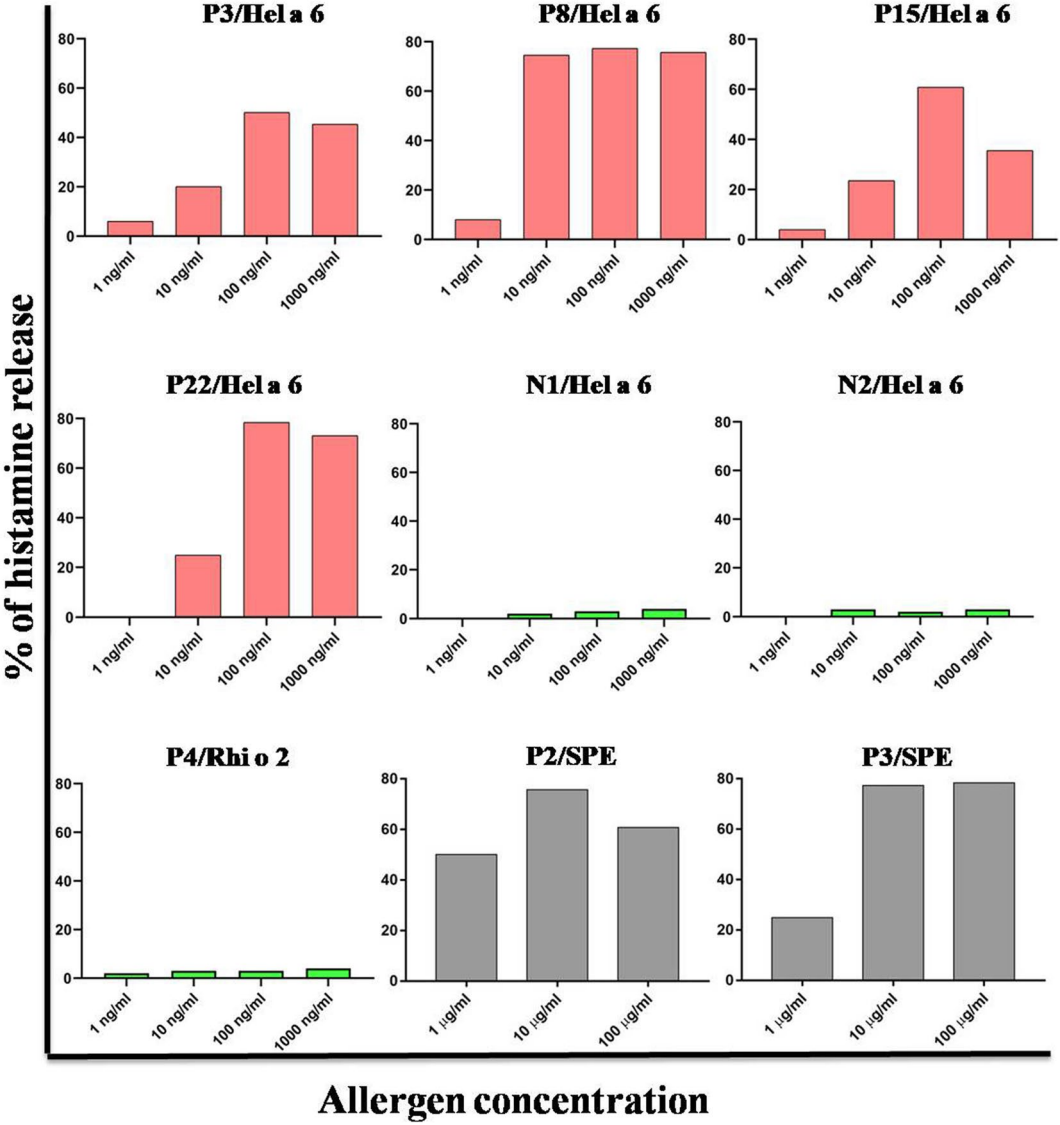
Hel a 6 displayed allergenic activity. Stimulation of effector cells in the blood of 4 sunflower-sensitized patients (red bars) with increasing concentrations of purified Hel a 6 (*x*-axis) resulted in mediator release (*y*-axis). For negative controls (green bars), either non-atopic bloods (N1 and N2) were stimulated with Hel a 6 or Hel a 6-specific atopic blood (P4) were stimulated with an unrelated allergen Rhi o 2. For positive control (gray bars), Hel a 6-specific atopic bloods (P2 and P3) were stimulated with sunflower pollen extract i.e. SPE. As it is a crude extract, hence, the protein concentration in extract was 10 times higher in for each of the 3 doses as compared to purified allergens.

In addition to IgE-reactivity, the allergenic activity of the purified Hel a 6 was studied by histamine release assay by active sensitization method. The assay was performed with peripheral blood collected from 4 sunflower-allergic patients with significantly elevated specific IgE-level for Hel a 6 and clinically active pollinosis symptoms. Effector cells in the blood samples were stimulated with an increasing concentration of purified Hel a 6. The cut-off value for release was set as 5%. As shown in the dose-dependent curves in Fig. 3, histamine release started to take place at 10 ng/ml concentration of Hel a 6, and the optimal release was found at 100 ng/ml of allergen. The release either declined or remained the same with a further increase of Hel a 6 concentration. The overall percentages of histamine release upon various concentrations of Hel a 6 challenge were found to be within a range from 20.13% to 78.42%. Expected results were observed in control experiments for histamine release.

### Hel a 6 displays pectate lyase activity

**Figure 4:**
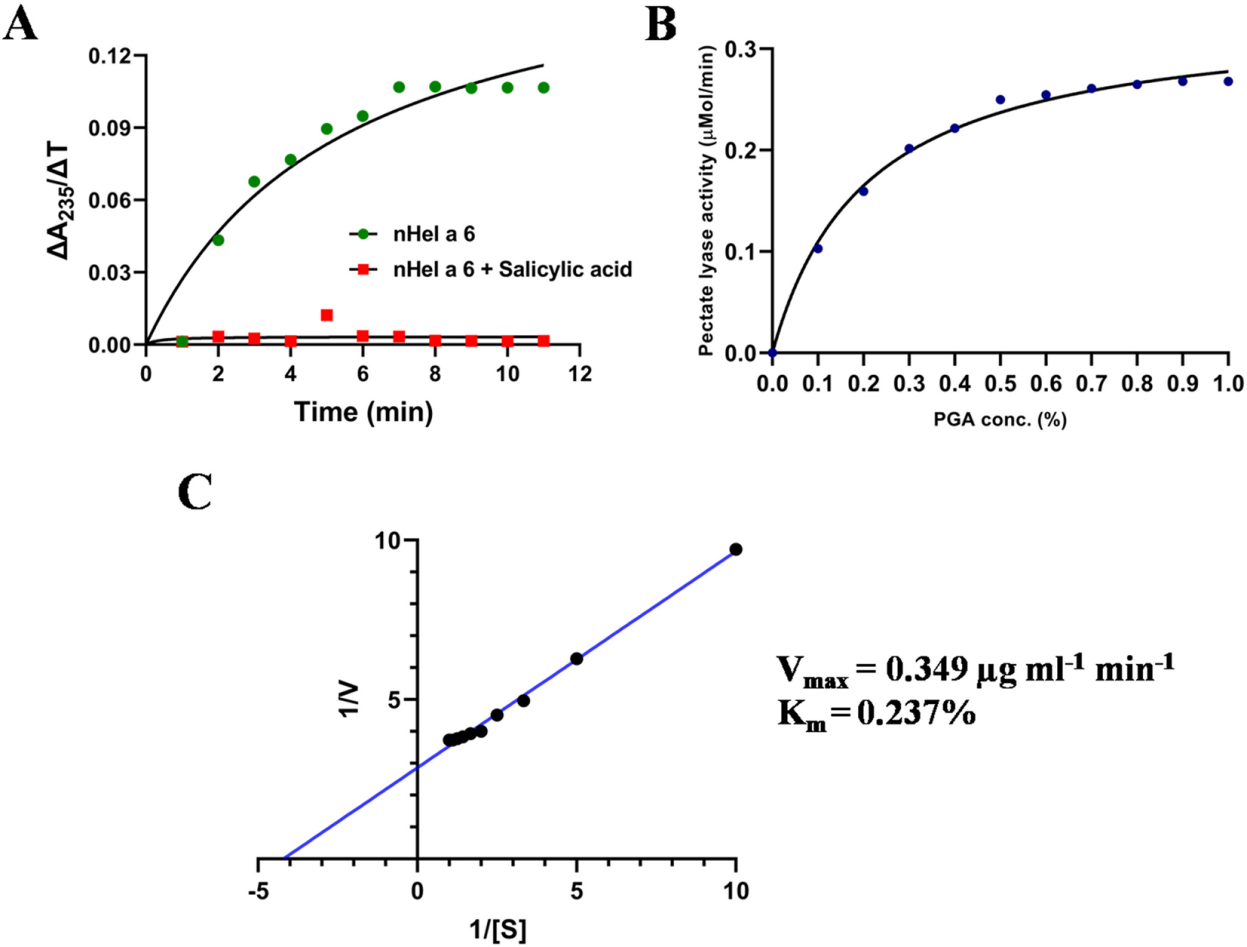
Hel a 6 is a pectate lyase. Pectate lyase activity is monitored by recording the cleavage of polygalacturonic acid (PGA) substrate by Hel a 6 and a concomitant increase in A_235_ of the reaction mix. (**A**) Rate of pectate lyase activity by Hel a 6 was determined at a fixed PGA concentration and expressed as a ratio of change in A_235_ and change in incubation time (ΔA_235_:ΔT; *y*-axis) as a function of time (minutes; *x*-axis). Addition of pectate lyase inhibitor salicylic acid resulted in a sharp decrease in enzyme activity. Kinetics of pectate lyase activity of Hel a 6 (*x*-axis) as a function of increase PGA concentration (*y*-axis) shown as non-linear Michaelis–Menten curve (**B**) and linear double reciprocal Lineweaver Burk plot (**C**) to determine the V_max_ and K_m_.

The sequence information of Hel a 6 revealed the presence of a putative pectate lyase domain as analyzed in the Pfam database. Hence, the biological activity of Hel a 6 was verified by in vitro enzyme assay using pectin or polygalacturonic acid (PGA) as a substrate and Ca^2+^ as a cofactor as described previously^9^. The assay is based on the principle that pectate lyase splits the glycosidic bonds of d-galacturonic acid by the mechanism of P-elimination to produce unsaturated uronides. The reaction was allowed to take place at 40 °C and pH 8.0, as previously determined to be the optimum for pectate lyase activity^10^. In the first set of reactions, the rate of pectate lyase activity of Hel a 6 was monitored using a fixed substrate concentration (i.e., 0.2% of PGA) as a function of time. It was observed that the rate of enzyme activity increased over time and reached the saturation level after 6 min indicating the optimal time for the enzyme–substrate reaction (Fig. 4A). In contrast, the enzyme activity was ceased entirely when salicylic acid was added to the reaction mix, which was already reported as a pectate lyase inhibitor^10^. In the second set of reactions, the enzyme assay was performed with increasing substrate concentration (0–1% of PGA) for 6 min, which showed a typical Michaelis–Menten form of enzyme kinetics (Fig. 4B). For the determination of kinetics data of Hel a 6, a double reciprocal linear plot (Fig. 4C) was generated. The V_max_ and K_m_ of the PGA-saturated Hel a 6 were determined to be 0.349 µMol/min and 0.237%, respectively.

### Folding pattern of Hel a 6

**Figure 5:**
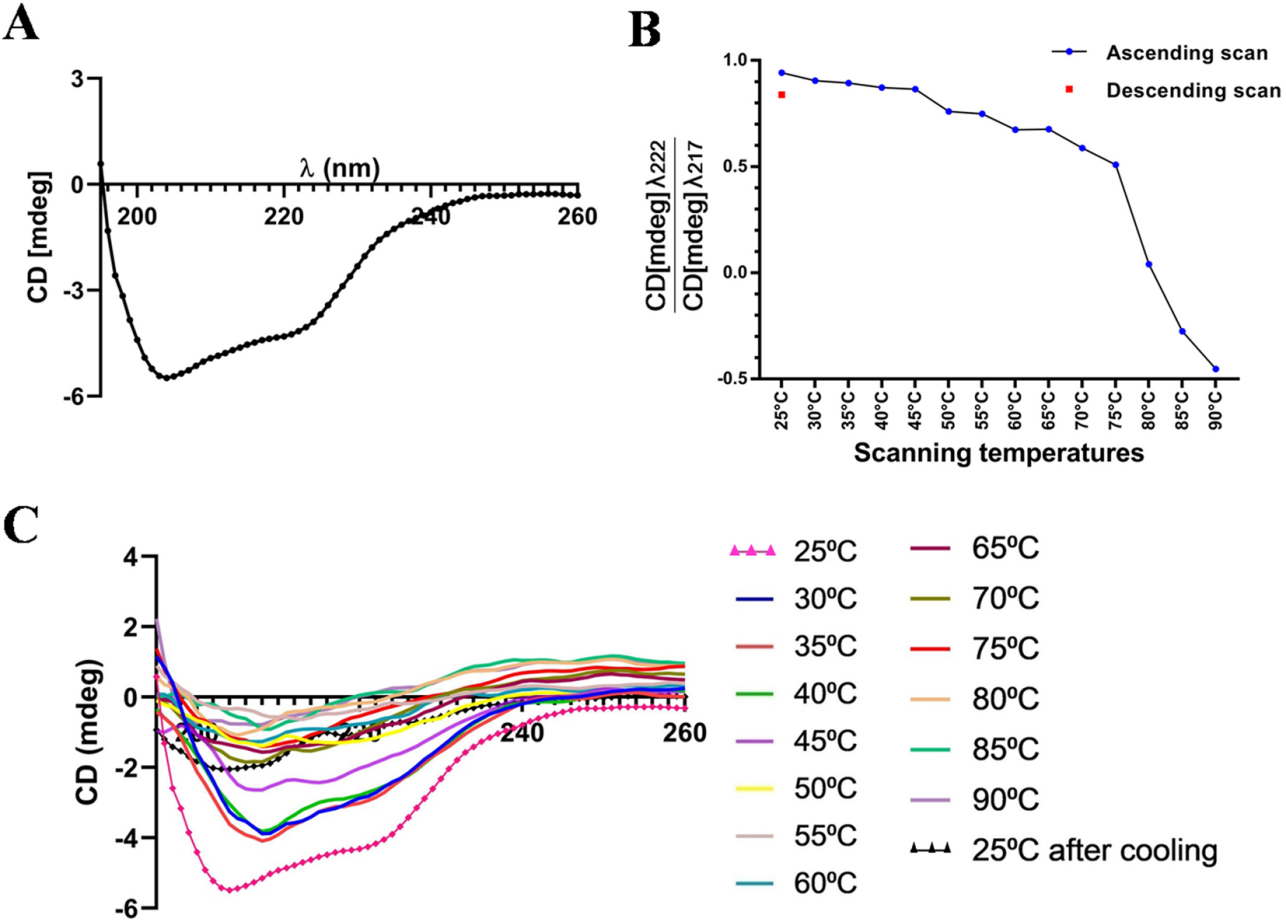
Folding pattern of Hel a 6. (**A**) CD spectra of 4 µM of Hel a 6 showing the CD milli degree (y-axis) at different wavelengths (nm; *y*-axis). (**B**) Melting curve of Hel a 6 showing the ratio of CD spectra at 222 nm and 217 nm taken at increasing temperatures (up scan) to a fully denatured state followed by decreasing the temperature up to 25 °C (down scan) to allow refolding of the protein. (**C**) Thermal denaturation of Hel a 6 showing the entire CD spectra (y-axis) as a function of various wavelengths (*x*-axis) taken at various temperatures.

**Figure 6:**
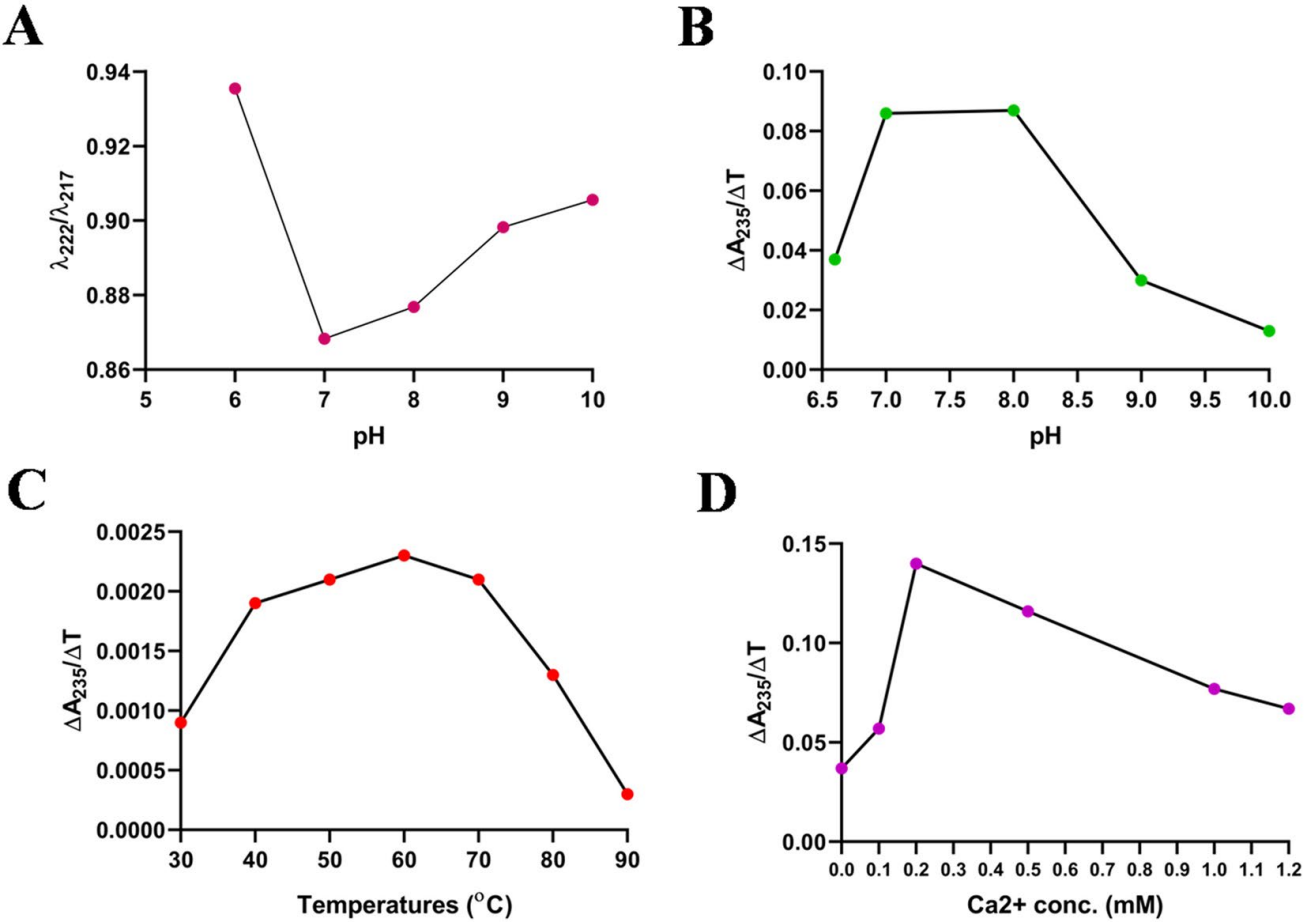
Effects of various parameters on the folding and activity of Hel a 6. (**A**) Effect of pH (*x*-axis) on the folding pattern of Hel a 6 (λ_222_:λ_217_) in y-axis. Effects of pH (**B**), temperature (**C**), and calcium concentration (**D**) plotted in y-axis, and the rate of enzyme activity plotted on *x*-axis.

Far-UV circular dichroism (CD) spectra of the purified Hel a 6 at 25 °C indicated that the purified allergen was in a properly folded state (Fig. 5A). The protein showed an almost equal mixture of α-helices and β-sheets, with α-helical characters being most predominant. The minimum was obtained at 217 nm, along with a characteristic shoulder at 222 nm. The refolding behavior of Hel a 6 was studied by generating a melting curve in which the CD spectra of Hel a 6 were recorded at increasing temperatures. As shown in Fig. 5B, C, the protein started to unfold at 45 °C and got completely denatured after 85 °C. Transition temperature (T_m_) was obtained at 55 °C. However, the protein partially refolded and regained ~ 80% of its native folds after re-cooling the system, indicating a reversible pattern of denaturation. In addition to temperature, the effect of pH on the secondary structure elements of Hel a 6 was studied in which the CD spectra were recorded at pH ranging from 6.0 to 10.0. However, very little change in secondary structural elements was observed with respect to pH (Fig. 6A).

### Effect of pH, temperature, and Ca^2+^ concentration on pectate lyase activity of Hel a 6

The enzyme activity of Hel a 6 at a fixed substrate concentration was recorded as a function of pH (Fig. 6B). The enzyme activity increased over pH, starting from 6.5 and then optimum activity was observed until pH 8.0, after which a sharp decline in the enzyme activity was observed. This typical bell-shaped curve suggested a functional instability of Hel a 6 at basic pH. Significantly reduced activity was observed at pH < 6.0. Hence, for subsequent studies, enzyme assays were performed at pH 8.0. The effect of temperature on the pectate lyase activity of Hel a 6 was studied by step-scan spectrophotometry, as shown in Fig. 6C, which revealed a gradual increase in the enzyme activity of Hel a 6 after 30 °C and the optimum activity was achieved at 60 °C. A gradual decrease in the activity was observed after further increasing the temperature. The effect of Ca^2+^ on pectate lyase activity is shown in Fig. 6D. The enzyme showed its highest activity at 0.2 mM Ca^2+^ concentration. A significant decrease in enzyme activity was observed in the presence of 1 mM EDTA, indicating the Ca^2+^ requirement of Hel a 6 for its functional activity (data not shown).

### Sequence conservation of Hel a 6 with other pectate lyases

**Figure 7:**
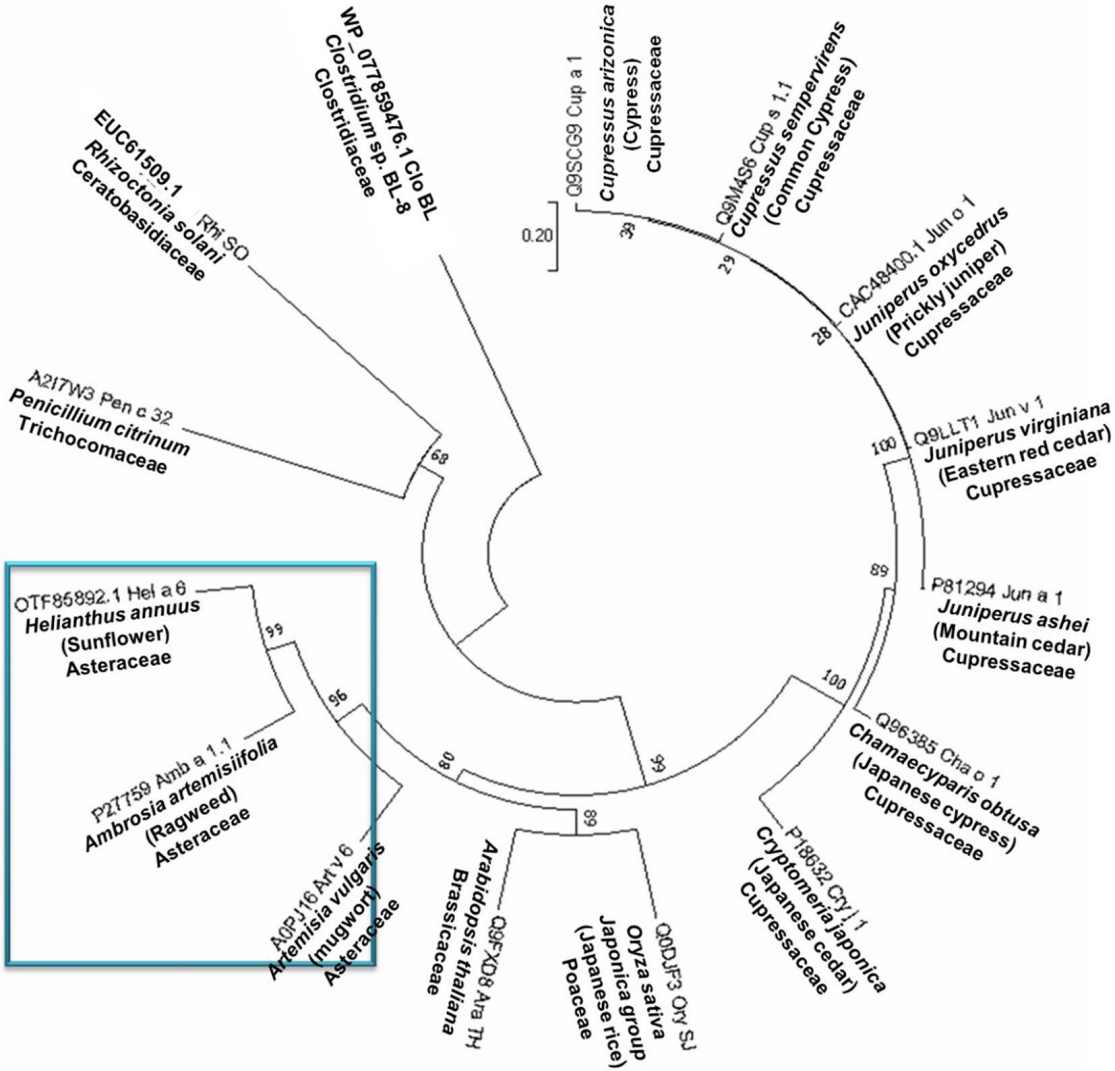
Evolutionary relationship among the pectate lyases. The unrooted phylogenetic tree constructed with 10 allergenic pectate lyases of plant origin enlisted in IUIS allergen database along two non-allergenic pectate lyases from rice and *Arabidopsis*, and two pectate lyases of fungal origin using MEGA v7.0 software (https://www.megasoftware.net). A separate clad from by Hel a 6, Amb a 1, and Art v 6 are marked within a blue box.

A multiple sequence alignment (Supplementary Fig. S1) of 10 pectate lyase allergens enlisted in the IUIS allergen database showed a substantial level of sequence conservation among pectate lyase allergens within Asteraceae and Cupressaceae family. The percentage of sequence identity between different pectate lyase allergens were represented in Supplementary Table S2. However, the sequence conservation was less between the members of two families as they formed different clades in the phylogenetic tree constructed on the basis of evolutionary relationship. Asteraceae members are closely related to non-allergenic pectate lyases from rice and *Arabidopsis thaliana*. On the contrary, Pen c 32, a fungal pectate lyase allergen from *Penicillium,* formed a completely separate branch with the non-allergenic pectate lyase from *Rhizoctonia solani*. The unrooted phylogenetic tree of pectate lyases and the pair-wise distance used to calculate the evolutionary relationship among them based on the amino acid sequences is shown in Fig. 7. As observed in the phylogenetic tree, Hel a 6 formed a separate cluster with Amb a 1 from ragweed and Art v 6 from mugwort, suggesting a high probability of cross-reactivity among them.

### Hel a 6 shows cross-reactivity with homologous allergens from ragweed and mugwort

**Figure 8:**
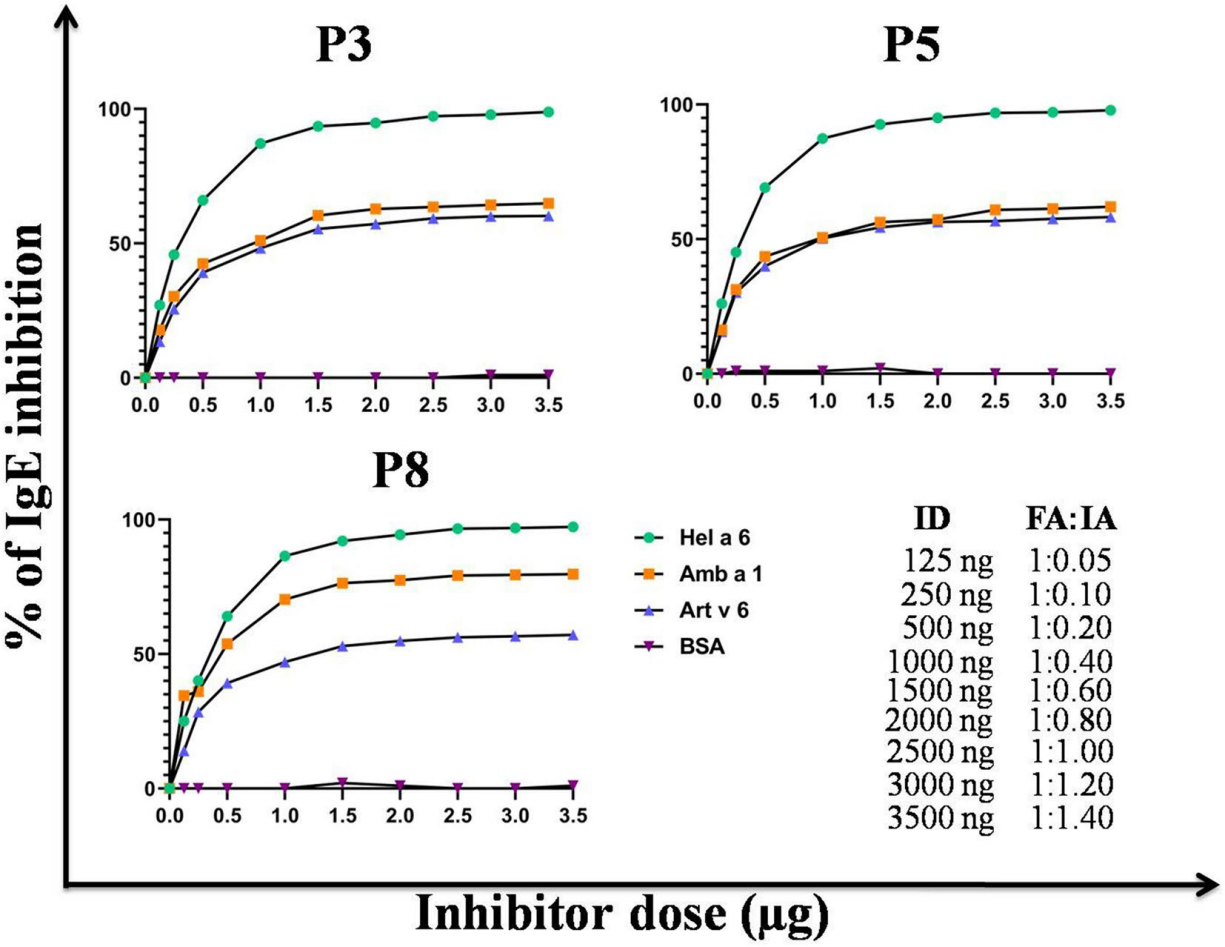
Cross-reactivity by ELISA inhibition. ELISA inhibition in which the plate-bound 0.5 µg/ml Hel a 6 was exposed to sera from 3 sunflower-sensitized patients pre-incubated with increasing doses (x-axis) of either Art v 6 or Amb a 1 as fluid phase inhibitors. Sera were also pre-incubated with either Hel a 6 (auto-inhibition) or BSA (negative control). The percentage of IgE-binding inhibition was plotted on *y*-axis. Lower right panel shows the ratio between fluid phase allergen (FA) and plate-bound immobilized allergen (IA) against each of the inhibitor doses (ID).

**Figure 9:**
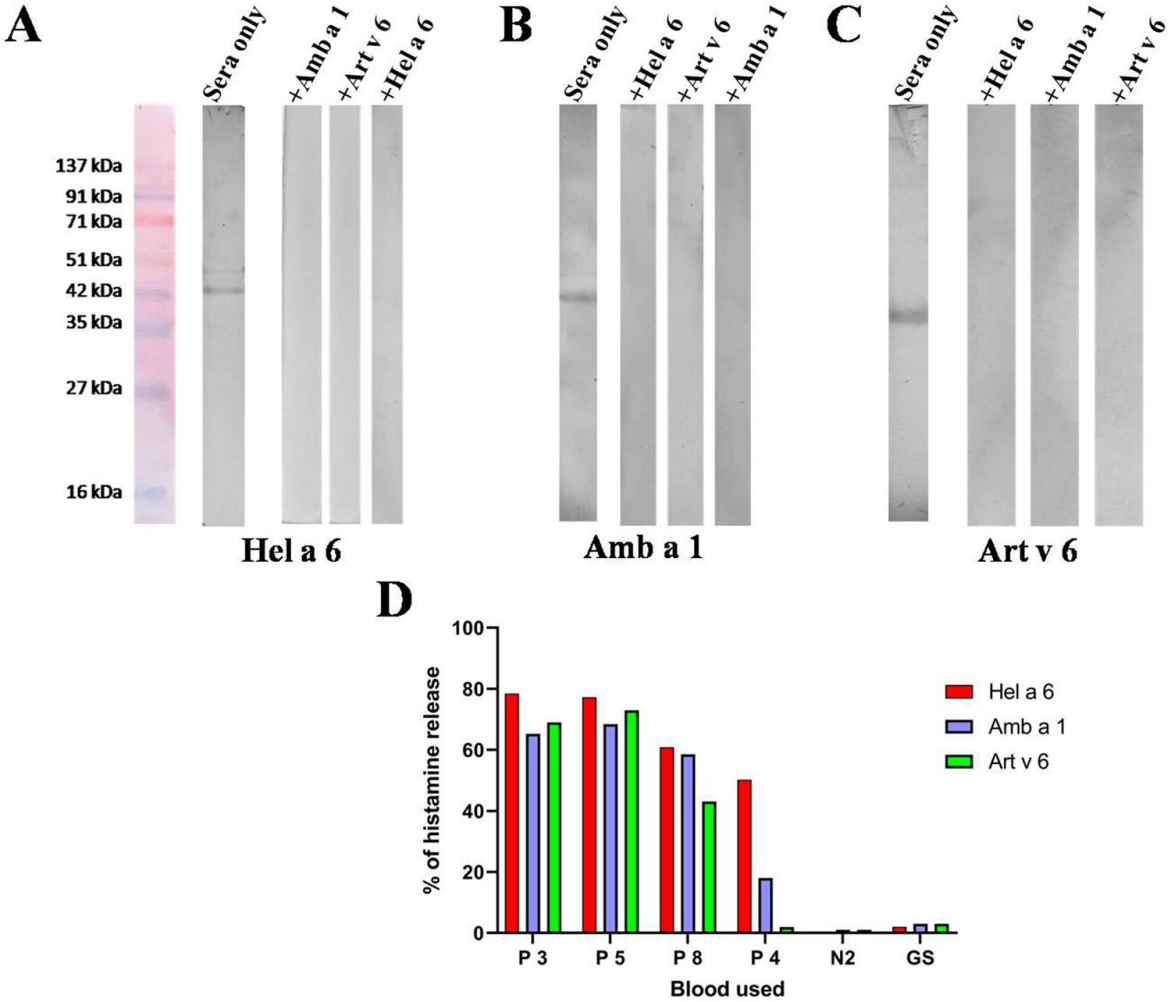
Cross-reactivity by blot inhibition and cross-stimulated mediator release. Hel a 6 (**A**), Amb a 1 (**B**), and Art v 6 (**C**) transferred on PVDF membrane were exposed to a pool of sunflower-sensitized sera pre-incubated with 10 µg/ml of either the same allergen (auto-inhibition) or the other two pectate lyases. Immunoblot was also done with only sera (i.e., no pre-incubation) or negative control. (**D**) Histamine release (*y*-axis) was observed when the effector cells in the blood of 4 sunflower-sensitized patients (*x*-axis) were stimulated with optimal concentration (100 ng/ml) of either Hel a 6 or Amb a 1 or Art v 6. For negative controls, bloods from healthy donor (N2) and grass pollen allergic sera (GS) were used.

Cross-reactivity of Hel a 6 with the purified natural Amb a 1 and Art v 6 was studied by ELISA inhibition and immunoblot inhibition experiments. Out of 10 Hel a 6 positive patients, 6 patients showed IgE-reaction with purified Amb a 1 and Art v 6, as shown in Supplementary Table S3. Sera from 3 such sunflower-sensitized patients (P3, P5, and P8) with high levels (P/N ~ 4.0) of specific IgE-titer against Amb a 1 and Art v 6 were used for IgE-ELISA inhibition assays. Different extents of IgE-inhibitions were observed when the plate-bound Hel a 6 (solid phase) was exposed to patient serum pre-incubated with either Amb a 1 or Art v 6 as a fluid phase inhibitor. For the 3 patients, a maximum of 57–65% and 60–80% of IgE-inhibition were observed for Amb a 1 and Art v 6, respectively. Maximum inhibitions were achieved at a dose of 1.5 µg of each of the 2 inhibitor allergens. The inhibition data are illustrated in Fig. 8. For immunoblot inhibition, pooled sera of 3 sunflower-sensitized patients were pre-incubated overnight either with Amb a 1 or Art v 6 or Hel a 6. Sera mixed with either Art v 6 or Amb a 1 displayed complete inhibition of IgE-binding to Hel a 6 on PVDF membrane in immunoblot as compared to the uninhibited control using sera without any pre-incubation (Fig. 9A). For auto-inhibition control, Hel a 6 on the membrane was exposed to sera pre-incubated Hel a 6 which resulted in full inhibition of IgE-binding (Fig. 9A). In 2 reciprocal experiments (Fig. 9B, C), Amb a 1 and Art v 6 on the PVDF membrane were exposed to allergen pre-incubated sera. In all the cases, complete inhibition of IgE-binding was observed to the membrane-bound allergens.

### Art v 6 and Amb a 1 cross-stimulated histamine release from Hel a 6-allergic patients

In addition to IgE-cross reactivity, the ability of Amb a 1 and Art v 6 to cross-stimulate the effector cells of Hel a 6-allergic patient was studied by histamine release assay (Fig. 9D). The basic principle of this assay is the same as already described in the previous section with an exception that the 4 Hel a 6-allergic blood samples were now stimulated with either Art v 6 or Amb a 1. About 100 ng/ml of each of these two allergens was used for the challenge as already determined to be the optimum concentration for maximum release with Hel a 6. Upon cross-stimulation with Amb a 1 and Art v 6, histamine release was observed within a range from 58%—62% and 43% -72% for the 4 patients.

### Mapping of putative cross-reactive epitopes of Hel a 6, Amb a 1, and Art v 6

**Figure 10:**
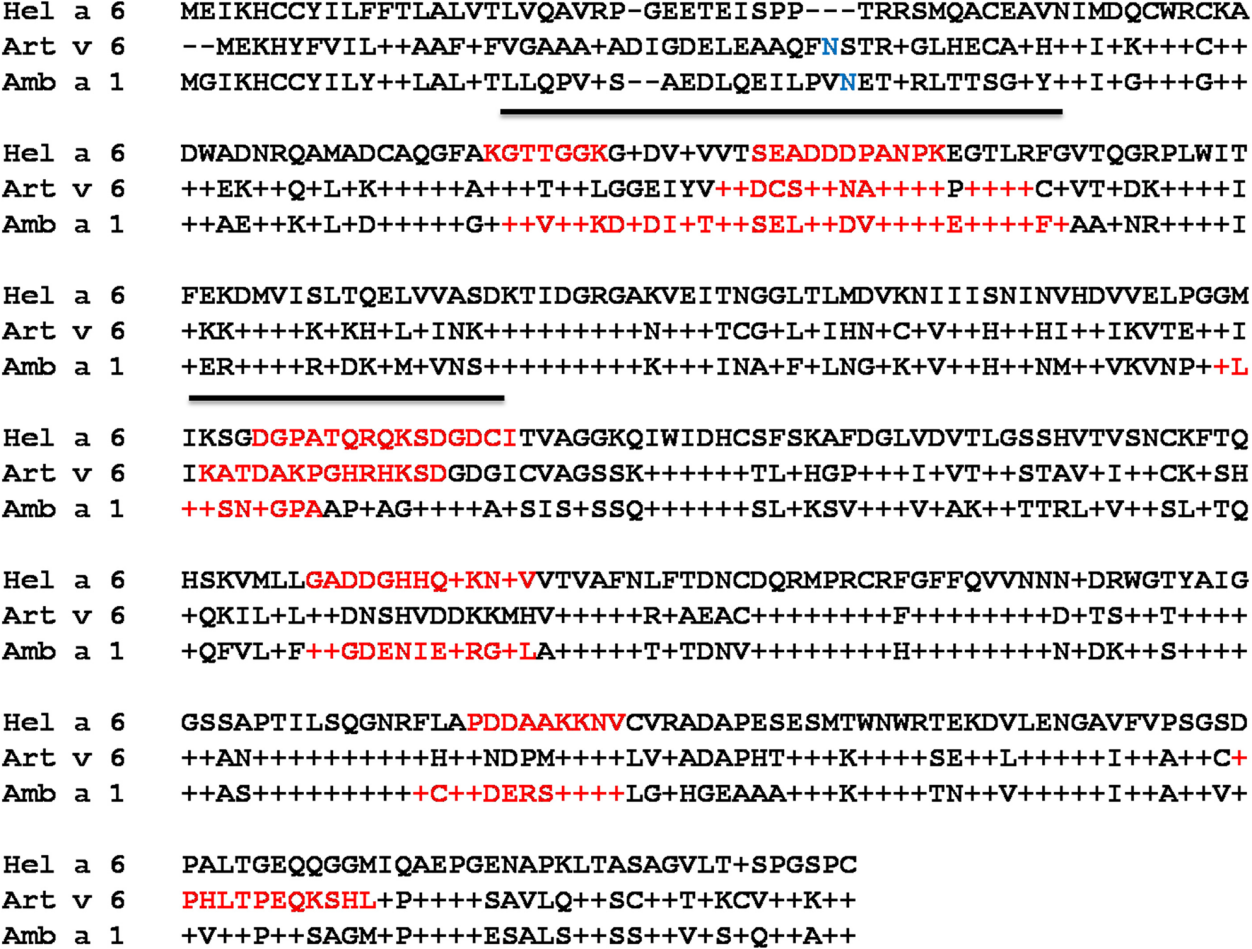
Sequence conservation within common pectate lyase allergens of weed origin. Multiple sequence alignment of Hel a 6 as reference sequence with 2 pectate lyases of Compositae family such as Art v 6 from mugwort and Amb a 1 from ragweed as aligned sequences. Identical residues and gaps in the aligned sequences are shown + and − respectively. Putative linear B cell epitopes are in red and N-linked glycosylation sites in blue. The regions containing B cell epitopes commonly present in all the 3 allergens are marked with a line that can be anticipated as the possible IgE-epitope responsible IgE-cross reactivity.

**Figure 11:**
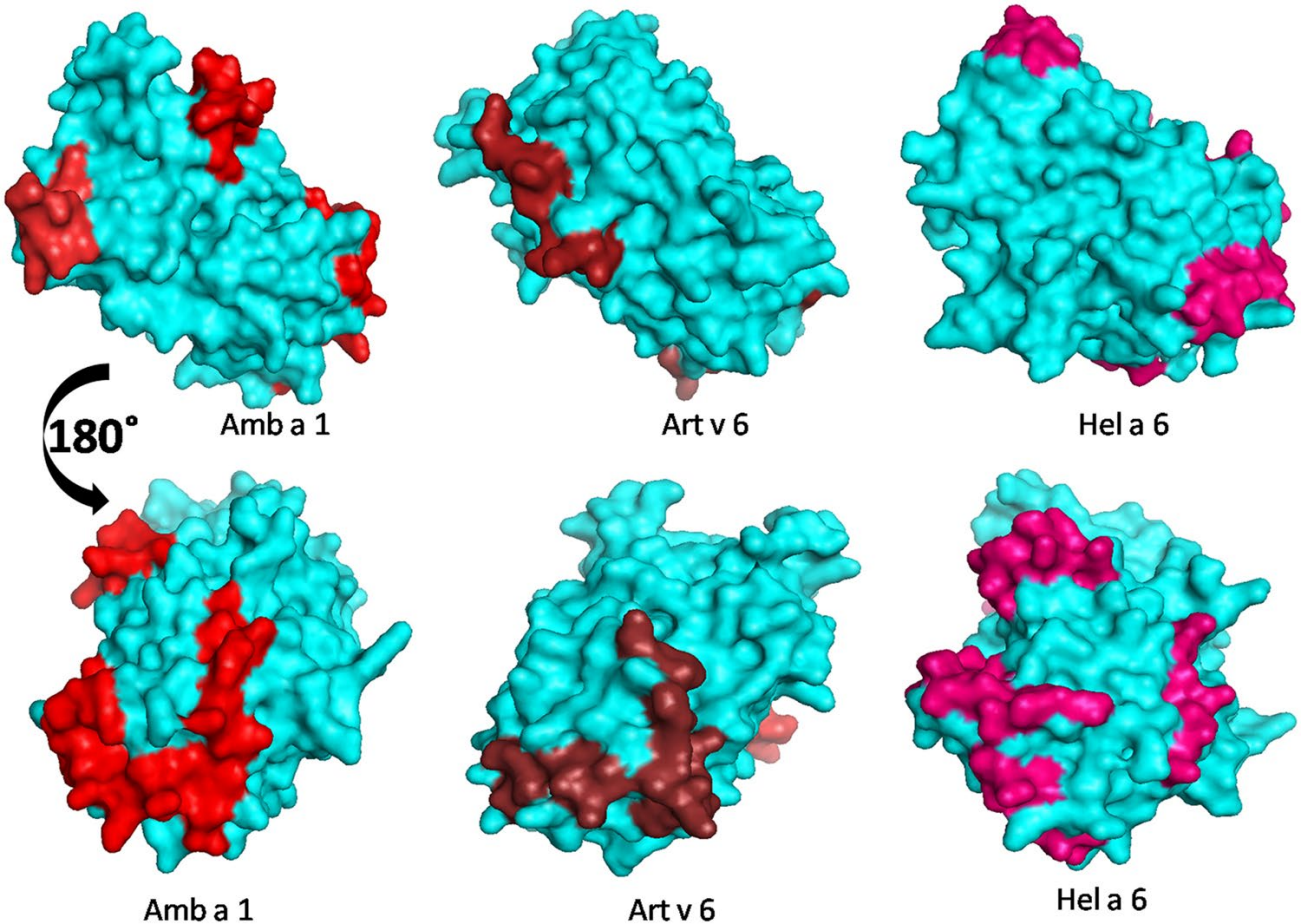
In silico Epitope mapping. The putative linear B cell epitopes (different shades of red) predicted in this study are mapped on the globular/surface representation of the corresponding 3D models (in cyan) of Amb a 1 (**A**), Art v 6 (**B**), and Hel a 6 (**C**) using PyMol v1.74 (https://pymol.org/academic).

A multiple sequence alignment (Fig. 10) with these 3 allergens revealed Hel a 6 having 68% and 63% sequence homology with Amb a 1 and Art v 6, respectively. Linear B cell epitopes of these allergens were predicted using ABCPRED and BCEPRED servers. The commonly identified epitopes by both the server having a high score in ABCPRED and supported by multiple physio-chemical parameters in BCEPRED were preliminarily selected as putative epitopes. Further filtering was done based on surface exposure, sequence conservation, and the presence of critical residues for IgE binding. A total of 5, 4, and 3 linear B cell epitopes were finally selected for Hel a 6, Amb a 1, and Art v 6 respectively. Predicted B cell epitopes were marked in the multiple sequence alignment (Fig. 10), which showed a substantial degree of conservation. As shown in the alignment, 2 of these epitopes were found to be shared by all the 3 allergens forming antigenic patches that might be crucial for IgE-cross reactivity among the pectate lyases of Asteraceae origin. The locations of these epitopes were mapped on the 3D structural models of the respective allergen in Fig. 11.

## Discussion

The present study describes the comprehensive characterization of a new major allergen of sunflower pollen designated as Hel a 6, which is a pectate lyase. After the sunflower profilin Hel a 2^11^, Hel a 6 is the allergen that has been purified and biochemically characterized in this study. Pectate lyases have long been reported as allergens from various weeds of the Asteraceae plant family^2,3,12^. Discovery of Hel a 6 is a new addition to this meagre list. Purified Hel a 6 displayed 57% IgE-reactivity within the sunflower-allergic patient population suggesting it to be a major sunflower allergen. Sero-reactive IgE-antibodies against Hel a 6 was observed in patients with typical outdoor respiratory symptoms of pollinosis and rhinitis. This observation tends to indicate that primary sensitization with Hel a 6 might have taken place via the route of inhalation. Hel a 6 is also clinically relevant as it was able to induce degranulation of the effector cells. Therefore, considering the fact that Hel a 6 is a clinically relevant major sunflower allergen, it can be a potential candidate for the diagnosis of pollinosis and occupational allergy. Pectate lyases are reported to have N-linked glycosylations, which in many cases, were reported to serve as cross-reactive carbohydrate determinants (CCDs). However, no such potential site for N-linked glycosylation was predicted in silico for Hel a 6. Also, we could not find any clinching experimental evidence on the presence of glycosylation motif in Hel a 6 through either periodic acid Schiff (PAS) staining or PNGaseF digestion (data not shown). This observation has strengthened the relevance of peptide epitopes for Hel a 6. Hel a 6 was found to be a monomer similar to the other reported pectate lyases and was functionally active. The Ca^2+^ requirement for the pectinolytic activity of Hel a 6 confirmed this allergen as a pectate lyase but not a pectin lyase^13^. It was also observed that Hel a 6 remained functionally steady and retained most of its native structural folds even under a broad range of physicochemical conditions such as pH and temperature. Such structural stability is the characteristic property of many respiratory allergens that remain structurally stable in various microenvironments and sensitize the respiratory mucosa in a globular form. The melting curve of Hel a 6 and its IgE-reactivity in immunoblot under denaturing condition led us to infer that IgE-binding to this allergen could be mediated by conformational epitopes of the allergen refolded on the PVDF membrane during the western blot. Conformational IgE-epitope was also reported in Amb a 1, the ragweed homolog of Hel a 6. Hel a 6 shared > 60% conserved sequences and structural folds with Amb a 1 and Art v 6. All these 3 allergens were found to have a characteristic β-barrel in the core surrounded by α-helices and short β-strands. This substantial level of structural similarity was substantiated by the presence of IgE-cross reactivity among these 3 allergens. However, it is also noteworthy to mention here that the patient cohort in this study did not have primary sensitization with either Amb a 1 or Art v 6 since both of these weeds are not commonly grown in the city where the present study was conducted. Hence, these patients were originally sensitized to Hel a 6, and therefore IgE-reactivity was not simply due to cross-sensitization. A further detailed study on the other pectate lyase allergens from local weeds and experimental comparison of their allergenic potential is warranted for a better diagnostic purpose. In this study, we also took an in silico approach for predicting the putative IgE-epitopes. A number of linear B cell epitopes were predicted from each of these cross-reactive pectate lyases. Out of them, we found 2 such epitopes commonly shared by these 3 allergens. Hence, it can be anticipated that these 2 epitopes are possibly responsible for cross-IgE reactivity among Hel a 6, Amb a 1 and Art v 6.

Interestingly, despite being continuous in nature, it was also observed that these 2 epitopes were presented in a conformational manner on the surface of each of these 3 allergens. This observation corroborates well with our previous idea on conformational epitope mediated cross-reactivity. However, structural data is needed to validate our in silico prediction. Taken together, Hel a 6 is a potential candidate molecule for the component-resolved diagnosis, and the purified allergen is a potential candidate for the therapy of weed-allergic patients.

## Materials and methods

### Pollen sampling

Pollen grains of cultivated sunflower were collected from fresh anthers, and 200 µ mesh was used to remove the non-pollen contaminants.

### Human subjects

Patients suffering from pollinosis, atopic rhinitis, and various other outdoor respiratory symptoms were skin tested with the pollen extracts of *Helianthus annuus*. About 70% of these patients were reported to have the cultivated sunflower farmlands located in their surroundings. Patients were then selected based on case history, positive cutaneous reaction, and specific-IgE level against the crude antigenic extract of sunflower pollen. Residual blood from 39 such patients and 6 healthy volunteers were collected with informed written consent. The study was approved by the human ethics committee of Bose Institute (BIHEC/2014-15/4) following Helsinki guidelines. Demographic and clinical details of the participants are enlisted in Supplementary Table S1.

### Protein extraction

After defatting with diethyl ether, around 1.2 g of pollen was crushed in liquid nitrogen and incubated in 10 ml of 20 mM Bis–Tris (pH 6.5) buffer containing 3% glycerol at 4 °C for overnight with shaking. After centrifugation at 7500*g*, the supernatant was sterilized through 0.22 µ PVDF membrane (Durapore Membrane Filters, Merck). The protein concentration was measured by Bradford assay using Bio-Rad Protein Assay Dye Reagent Concentrate (Bio-Rad) following the manufacturer's protocol.

### Column chromatography

All the chromatographic procedures were performed using an ÄKTAprime plus (GE Healthcare Life Sciences) FPLC system^14^. The crude pollen extract (5 mg/ml) was fractionated in HiTrap Q HP column (GE Healthcare Life Sciences) equilibrated with the same buffer followed by elution of column-bound proteins using a linear gradient of 0.1–1 M NaCl. Eluted fractions containing the desired protein at a higher level of purity were pooled, dialyzed against 20 mM phosphate buffer (pH 7.4), and then concentrated in < 10 kDa cut-off Amicon Ultrafiltration device (Millipore). About 2 mg of concentrated protein was loaded onto a Superdex 75 10/300 GL gel filtration column (GE Healthcare Life Sciences)^15^. Eluted fractions were screened by IgE-western blot using serum of 4 sunflower-sensitized patients to check for the presence of Hel a 6. The fractions containing Hel a 6 at > 90% purity were pooled, dialyzed against 1 M ammonium bicarbonate buffer (pH 8.5), and lyophilized.

### Mass spectrometry

As described in Ref.^16^, 50 µg of Hel a 6 was dissolved in 10 µl of denaturation buffer containing 6 M urea and 2 M thiourea in 10 mM HEPES buffer (pH 8.0). The sample was reduced by adding 1 µl of 10 mM DTT and alkylated by adding 1 µl of 55 mM iodoacetamide. Digestion was carried out with 1 µg of Lys-C Mass Spec Grade (Promega) from 0.5 µg/µl stock for 3 h at room temperature. The reaction mixture was then 4 times diluted, followed by the addition of 1 µg of Trypsin Gold, Mass Spectrometry Grade (Promega) and then incubated overnight at room temperature. Digestion was stopped by acidifying the sample to pH < 2.5 with 100% TFA^17^. The resultant peptides were purified using Pierce C18 Tips (Thermo Fisher Scientific) following manufacturer’s protocol. Around 10 µl of the sample was aspirated into the C18 tip. For the maximum binding of peptides, the process was repeated for 8–10 cycles. For MALDI TOF/TOF, the peptides were eluted with 2–10 µl of 0.1% TFA in 95% ACN and then coated onto MALDI plate with matrix. For LC–MS/MS, peptides were eluted with 2–10 µl of 0.1% formic acid in 95% ACN and then dispensed into auto-sampler vial^18^. MS/MS spectra were searched against NCBInr database using the parameters as mentioned in Table 1.

### Immuno-dot blot

Either 0.5 µg of purified Hel a 6 or 5 µg of sunflower pollen extract was spotted onto nitrocellulose membrane and blocked with 3% BSA. Strips were then exposed to sera of 39 sunflower-sensitized patients at 1:10 v/v at 4 °C for overnight. Bound IgE-antibodies were detected with Monoclonal Anti-Human IgE–Alkaline Phosphatase antibody produced in mouse (Sigma-Aldrich, Catalogue no. A3076) at 1:1000 v/v and NBT-BCIP substrate (Abcam).

### IgE-ELISA

MicroWell 96-Well Microplates (Nunc, Thermo Fisher Scientific) were coated with either nHel a 6 (5 ng/μl) or sunflower pollen extract in a bicarbonate buffer (pH 9.2) followed by blocking with 3% BSA (Sigma). Wells were then exposed to patient sera in 1:5 v/v at 4 °C for 16 h. Bound IgEs were detected by Monoclonal Anti-Human IgE−Alkaline Phosphatase antibody produced in mouse (Sigma-Aldrich, Catalogue no. A3076) at 1:1000 v/v and *p*-Nitrophenyl Phosphate (pNPP) Liquid Substrate System (Sigma-Aldrich). The reaction was stopped with 3 N NaOH, and absorbance was measured at 405 nm. An individual patient serum having P/N value [ratio between A_405_ of a patient serum (P) and the mean of healthy controls (N)] more than 2.0 was considered as positive for that allergen.

### Histamine release assay

As described in Refs.^19,20^, heparinized blood from 4 Hel a 6-sensitized patients were diluted 7 times with PBS and then challenged with increasing concentrations of nHel a 6 at 37 °C for 1 h. For spontaneous release, blood samples were incubated without any allergen challenge. Blood samples were then centrifuged at 8000*g* for 5 min and the supernatant was collected. For total release, white blood cells were isolated following erythrocyte removal using 10× RBC Lysis Buffer Solution (HiMedia Laboratories) and the cells were lysed with 10% Triton X. For cross stimulation, the blood samples from Hel a 6-allergic patients were challenged with 100 ng/ml of either Amb a 1 or Art v 6 or Hel a 6 (positive control). Released histamine in the cell-free supernatant was then quantified by histamine assay kit (HISTAMINE EIA, Beckman Coulter Inc.) and the percentage was calculated as

$$\% \,of\,mediator\,release = \left[ {\frac{Induced\,release - Spontaneous\,release}{{Total\,release - Spontaneous\,release}}} \right] \times 100.$$

### Pectate lyase assay

As originally described in Ref.^9^, 2.0 ml of reaction mixture was prepared with 0.2% (w/v) polygalacturonic acid (PGA) (Sigma-Aldrich), 25 mM Tris–HCl buffer (pH 8.0), 0.2 mM CaCl_2_ and 4 mM Hel a 6. The activity was determined by monitoring the increase in A_235_ at 37 °C in a UV-1280 UV–Vis Spectrophotometer (Shimadzu). One unit of pectate lyase activity was defined as the amount of the enzyme required to form 1 µM of unsaturated uronide product minute^−1^ with an extinction coefficient of 4.6 mM^−1^ cm^−1^ at 235 nm^10,21^. The reaction time to reach the saturation point and the effect of 10 mM salicylic acid on the enzymatic activity was measured by using a fixed PGA concentration of 0.2%. Then enzyme kinetics was analyzed by performing the reaction with variable substrate concentration ranging from 0–1%. In a separate experiment, the same assay was performed either in a temperature range from 30 to 90 °C at a fixed pH 8 or in Tris–HCl buffer of pH ranging from 6 to 9 at optimum temperature^10^. The effect of Ca^2+^ ion on the activity of nHel a 6 was studied by adding CaCl_2_ to the assay mixture at varying concentrations ranging from 0–1 mM followed by measuring the activity.

### CD spectrometry

CD spectra of 4 μM of purified Hel a 6 in 5 mM NaH_2_PO_4_, and 2 mM NaCl (pH 7.4) was recorded in a J-815 circular dichroism (CD) spectropolarimeter (Jasco, Inc., MD, USA) at 25 °C within a wavelength range of 195–260 nm as described in Ref.^22^ and analyzed using CDNN software. The thermal melting of the protein was studied by recording the CD spectra at a temperature range of 25–90 °C with a heating rate of 1 °C min^−11^ and a scan speed of 50 nm/min (up-scan) followed by recording the spectra again after cooling down the system to 25 °C (down-scan). In a separate experiment, CD spectra were taken after dissolving Hel a 6 in phosphate buffer with pH adjusted from 6.0 to 10.0 at 25 °C. Ratios of the ellipticities at 222 nm and 217 nm were calculated and plotted as a function of either temperatures or pH.

### Bioinformatics studies

The sequence of Hel a 6 was aligned with 10 reported pectate lyase allergens enlisted in IUIS allergen database using MUSCLE v3.8 software. Phylogenetic analysis of these pectate lyases was performed by the Neighbour-joining method with 1000 bootstraps using MEGA v7.0 software (https://www.megasoftware.net)^23^. The pair-wise distance was calculated using Poisson model. A second alignment was also performed with Hel a 6, Amb a 1 and Art v 6.

### Purification of natural Amb a 1 and natural Art v 6

As previously described^5,24^, pollen extracts (Allergon, Thermo Fischer) in PBS buffer pH 7.2 (0.15 g/ml) were fractionated by ultrafiltration, followed by ion exchange and size exclusion chromatography. Homogeneity of purified allergens was verified by SDS-PAGE, CD spectroscopy, and mass spectrometry.

### ELISA inhibition

A pool of sera from 3 Hel a 6-reactive patients having P/N > 4.0 against Amb a 1 and Art v 6 were used for ELISA inhibition as described in Ref.^19^. Briefly, 0.5 µg/ml of plate-bound Hel a 6 was exposed to this serum pool (1:10 v/v) separately pre-incubated with either Amb a 1 or Art v 6 or Hel a 6 (auto-inhibitor) or BSA (non-inhibitor) at increasing doses for overnight at 4 °C and bound IgE-antibodies were detected. The percentage of IgE-inhibition was determined as

$$\left( {1 - \frac{OD405 \,of\,the\,sample\,with\,inhibitor}{{OD405\,of\,the\,sample\,without\,inhibitor}}} \right) \times 100.$$

### Immunoblot inhibition

Those 3 sera used in ELISA-inhibition were pooled and pre-incubated with 10 µg/ml of either Amb a 1 or Art v 6 or Hel a 6 (auto-inhibition). About 5 µg of Hel a 6 on PVDF membrane was exposed to these sera. In a reciprocal experiment, Amb a 1 and Art v 6 on PVDF membrane were probed with sera pre-incubated with Hel a 6. Uninhibited controls were prepared with sera without allergen pre-incubation, and bound IgE-antibodies were detected.

### In silico epitope mapping

The energy-minimized 3D structural models of Hel a 6, Amb a 1, and Art v 6 were built using the SWISS-MODEL server as described earlier^14^. Linear B cell epitopes were predicted in ABCpred^25^ and BCEPRED^26^. Peptides with residues above a threshold value of 2.38 in all the seven physicochemical scales of BCEPRED were selected^19^. ABCpred server was also run (threshold > 0.5) to compare and verify the results of BCEPRED analysis. The predicted epitopes were then mapped on the 3D model using PyMol v1.74 (https://pymol.org/academic).

### Statistical analysis

The specific IgE-levels in patient sera against crude extract and Hel a 6 were compared using Mann–Whitney U test, and correlation between them was analyzed by two-tailed non-parametric Spearman correlation analysis using Graphpad Prism v8. A p value < 0.05 was considered as statistically significant.

## Supplementary information

Supplementary Information.
