# Editorial Expression of Concern: Purification and biochemical characterization of Hel a 6, a cross-reactive pectate lyase allergen from Sunflower (*Helianthus annuus* L.) pollen

**DOI:** 10.1038/s41598-024-74241-7

**Published:** 2024-10-01

**Authors:** Nandini Ghosh, Gaurab Sircar, Claudia Asam, Martin Wolf, Michael Hauser, Sudipto Saha, Fatima Ferreira, Swati Gupta Bhattacharya

**Affiliations:** 1 Department of Microbiology, Vidyasagar University, Paschim Medinipur, India; 2 Department of Botany, Institute of Sciences, Visva-Bharati, Santiniketan, India; 3 Department of Biosciences, University of Salzburg, Salzburg, Austria; 4 Cell Therapy Institute, (SCI‑TReCS), Paracelsus Medical University (PMU), Salzburg, Austria; 5 Division of Bioinformatics, Bose Institute, Kolkata, India; 6 Division of Plant Biology, Bose Institute, Kolkata, India

Editorial Expression of Concern to: *Scientific Reports*10.1038/s41598-020-77247-z, published online 19 November 2020

The Editors are issuing an Editorial Expression of Concern for this Article.

After the publication of this Article concerns were raised regarding image overlap between Figure 9a (Hel a 6) and 9b (Amb a 1). The Authors are unable to provide raw images for further investigation. Readers are therefore urged to take caution when interpreting the content of this Article.

Gaurab Sircar agrees with this Editorial Express of Concern. Michael Hauser, Sudipto Saha, Fatima Ferreira, and Swati Gupta Bhattacharya did not respond to correspondence from the Publisher about this Editorial Express of Concern. The Publisher is unable to get the current email addresses of Claudia Asam and Martin Wolf. Nandini Ghosh has passed away.

