# Supplementary material for: Purification and biochemical characterization of Hel a 6, a cross-reactive pectate lyase allergen from Sunflower (Helianthus annuus L.) pollen
Source: Sci Rep. 2020 Nov 19;10:20177. doi: 10.1038/s41598-020-77247-z (PMC7677321; doi:10.1038/s41598-020-77247-z)
Supplement: Supplementary file 1 — Supplementary Information. [file 41598_2020_77247_MOESM1_ESM.doc]

Purification and biochemical characterization of Hel a 6, a cross-reactive pectate lyase allergen from Sunflower (*Helianthus annuus* L.) pollen

**Nandini Ghosh^1, 6^#*, Gaurab Sircar^2^#, Claudia Asam^3^, Martin Wolf^3,4^, Michael Hauser^3^, Sudipto Saha^5^, Fatima Ferreira^3^, Swati Gupta Bhattacharya^6^***

^1^Department of Microbiology, Vidyasagar University, West Medinipur, India; ^2^Department of Botany, Institute of Sciences, Visva-Bharati, Santiniketan, India; ^3^Department of Biosciences, University of Salzburg, Austria; ^4^Cell Therapy Institute, (SCI-TReCS), Paracelsus Medical University (PMU), Salzburg, Austria, ^5^Division of Bioinformatics, Bose Institute, Kolkata, India; ^6^Division of Plant Biology, Bose Institute, Kolkata, India

* Joint corresponding author: [swati@jcbose.ac.in](mailto:swati@jcbose.ac.in); [nandini.ghosh14@gmail.com](mailto:nandini.ghosh14@gmail.com) Tel.: +913323031129

#Equal contribution

**Keywords:** Hel a 6, Sunflower (*Helianthus annuus* L.), pollen, allergen, allergy, IgE, pollinosis, pectate lyase

**Running title:** Characterization of sunflower allergen Hel a 6

**Supplementary materials:** Table S1, S2, and S3. Figure S1.

**Table S1: Clinico-demographic features of human subjects enrolled for the study.**

| **Human subject** | **Age** | **Sex** | **Disorders** | **SPT with SPE (>3mm)** | **Total IgE**  **(kUA/L)** | **sIgE against SPE**  **(P/N)** | **Histamine content (nMol/L)** | **Vicinity to sunflower plantation** |
| --- | --- | --- | --- | --- | --- | --- | --- | --- |
| P1 | 34 | F | AR+ BA | +2 | 103 | 5.23 | 330.52 | Yes |
| P2 | 49 | M | U | +3 | 124 | 4.81 | 355.91 | Yes |
| P3 | 48 | F | AR+ ANG | +3 | 134 | 5.89 | 284.36 | Yes |
| P4 | 35 | M | SOB | +3 | 100 | 3.86 | 306.97 | Yes |
| P5 | 25 | F | CC | +2 | 119 | 5.78 | 370.53 | Yes |
| P6 | 24 | F | CC | +3 | 122 | 2.69 | 367.92 | Yes |
| P7 | 20 | F | AR+ BA  SOB | +3 | 127 | 3.86 | 370.29 | Yes |
| P8 | 16 | M | SR | +2 | 105 | 5.84 | 207.76 | Yes |
| P9 | 30 | F | CC | +3 | 105 | 3.22 | 309.03 | No |
| P10 | 50 | F | AR+ BA | +3 | 107 | 3.13 | 255.71 | Yes |
| P11 | 47 | M | SOB | +3 | 111 | 2.88 | 267.13 | Yes |
| P12 | 36 | M | AR+ BA | +3 | 110 | 2.94 | 299.98 | Yes |
| P13 | 37 | M | AR+ BA | +3 | 105 | 3.52 | 289.26 | No |
| P14 | 48 | M | SR | +3 | 120 | 2.58 | 276.31 | Yes |
| P15 | 26 | F | SR | +3 | 115 | 2.67 | 316.19 | Yes |
| P16 | 19 | F | SR | +3 | 126 | 2.78 | 309.28 | Yes |
| P17 | 32 | F | ANG | +3 | 121 | 2.65 | 305.32 | Yes |
| P18 | 34 | M | CC | +2 | 116 | 2.55 | 269.23 | Yes |
| P19 | 26 | F | CC | +2 | 112 | 2.53 | 257.84 | Yes |
| P20 | 50 | M | ANG | +2 | 102 | 2.61 | 261.35 | Yes |
| P21 | 37 | M | SR | +3 | 110 | 2.83 | 250.32 | Yes |
| P22 | 46 | F | AR | +2 | 98 | 2.6 | 246.55 | No |
| P23 | 50 | F | BA | +2 | 100 | 2.66 | 259.78 | Yes |
| P24 | 19 | F | AR+BA | +2 | 99 | 2.86 | 236.59 | Yes |
| P25 | 21 | M | CC | +2 | 101 | 5.85 | 245.89 | Yes |
| P26 | 24 | F | SR | +2 | 120 | 2.74 | 201.87 | Yes |
| P27 | 23 | M | AR | +2 | 112 | 2.57 | 206.35 | Yes |
| P28 | 35 | M | SOB | +2 | 105 | 4.51 | 212.54 | Yes |
| P29 | 45 | M | AR+SOB | +2 | 104 | 4.98 | 195.85 | Yes |
| P30 | 49 | M | ANG | +2 | 96 | 4.71 | 216.45 | Yes |
| P31 | 32 | F | CC | +2 | 123 | 3.12 | 206.54 | Yes |
| P32 | 34 | M | BA | +2 | 113 | 3.01 | 218.74 | Yes |
| P33 | 37 | F | AR+BA | +2 | 97 | 5.88 | 214.56 | Yes |
| P34 | 42 | F | SR | +2 | 110 | 5.95 | 196.98 | Yes |
| P35 | 47 | F | SR | +2 | 114 | 3.21 | 178.96 | Yes |
| P36 | 44 | M | AR | +2 | 121 | 3.56 | 186.32 | Yes |
| P37 | 48 | F | SOB | +2 | 109 | 3.06 | 178.01 | No |
| P38 | 30 | M | AR | +2 | 112 | 2.62 | 165.23 | No |
| P39 | 35 | M | AR+BA | +3 | 124 | 3.89 | 168.58 | Yes |
| N1 | 18 | M | NS | - | 50 | - | 10 | Yes |
| N2 | 20 | F | NS | - | 46 | - | 15 | Yes |
| N3 | 35 | M | NS | - | 40 | - | 12.6 | Yes |
| N4 | 30 | F | NS | - | 48 | - | 17.2 | Yes |
| N5 | 59 | M | NS | - | 30 | - | 10.8 | Yes |
| N6 | 55 | F | NS | - | 41 | - | 12 | Yes |

*Abbreviations: AR- Allergic Rhinitis, BA- Bronchial Asthma, SOB- Shortness of Breath, U- Urticaria, ANG- Angioedema, CC- Cough & Cold, SR- Skin Rash, NS- No Symptom, C1 –C6 –Non-atopic healthy subjects, SPE – sunflower pollen extract.

** The grading scale of positive SPT: +1 if wheal diameter is 3–5 mm, +2 if >6 mm, +3 if >6 mm along with 1-2 small pseudopods, and +4 is any reaction that is more pronounced than +3. SPT negative if wheal diameter is <3 mm,

**Table S2**: Percent Identity matrix of different pectate lyase allergens

|  | Amb a 1 | Art v 6 | Hel a 6 | Cha o 1 | Cry j 1 | Cup a 1 | Cup s 1 | Jun a 1 | Jun o 1 | Jun v 1 | Pen c 32 |
| --- | --- | --- | --- | --- | --- | --- | --- | --- | --- | --- | --- |
| Amb a 1 | 100 |  |  |  |  |  |  |  |  |  |  |
| Art v 6 | 57.87 | 100 |  |  |  |  |  |  |  |  |  |
| Hel a 6 | 67.77 | 63.01 | 100 |  |  |  |  |  |  |  |  |
| Cha o 1 | 44.59 | 45.95 | 46.76 | 100 |  |  |  |  |  |  |  |
| Cry j 1 | 44.86 | 46.49 | 46.76 | 78.88 | 100 |  |  |  |  |  |  |
| Cup a 1 | 43.53 | 45.73 | 44.35 | 83.92 | 78.20 | 100 |  |  |  |  |  |
| Cup s 1.1 | 44.08 | 44.90 | 45.18 | 83.38 | 77.66 | 95.10 | 100 |  |  |  |  |
| Jun a 1 | 44.63 | 45.45 | 46.01 | 85.56 | 78.75 | 94.82 | 95.91 | 100 |  |  |  |
| Jun o 1 | 43.53 | 44.90 | 45.18 | 84.97 | 77.93 | 95.91 | 95.37 | 97 | 100 |  |  |
| Jun v 1 | 44.63 | 45.73 | 46.28 | 85.01 | 78.47 | 94.28 | 96.19 | 96.46 | 96.46 | 100 |  |
| Pen c 32 | 26.77 | 28.25 | 27.51 | 28.52 | 27.78 | 28.52 | 227.78 | 28.15 | 27.78 | 28.15 | 100 |

Table S3: Specific IgE-titre of selected sunflower sensitized patient sera against Amb a 1 and Art v 6

| **Patient No.** | **Specific IgE against Amb a 1 *** | **Specific IgE against Art v 6 *** | **Specific IgE against Hel a 6 *** |
| --- | --- | --- | --- |
| 1 | 2.98 | 3.55 | 5.23 |
| 2 | 3.14 | 3.25 | 4.81 |
| 3 | **4.26** | **5.16** | **5.89** |
| 4 | **3.26** | **3.33** | **3.86** |
| 5 | **4.31** | **4.40** | **5.78** |
| 6 | 2.11 | 2.25 | 2.69 |
| 7 | 2.06 | 1.97 | 3.51 |
| 8 | **4.69** | **4.54** | **5.84** |
| 9 | 1.89 | 2.14 | 3.22 |
| 10 | 1.74 | 2.02 | 3.13 |

*Specific IgE values were given as a ratio of OD._405_ of patient sera in respect to control sera (P/N). P/N value> 2.5 were regarded as positive result

**
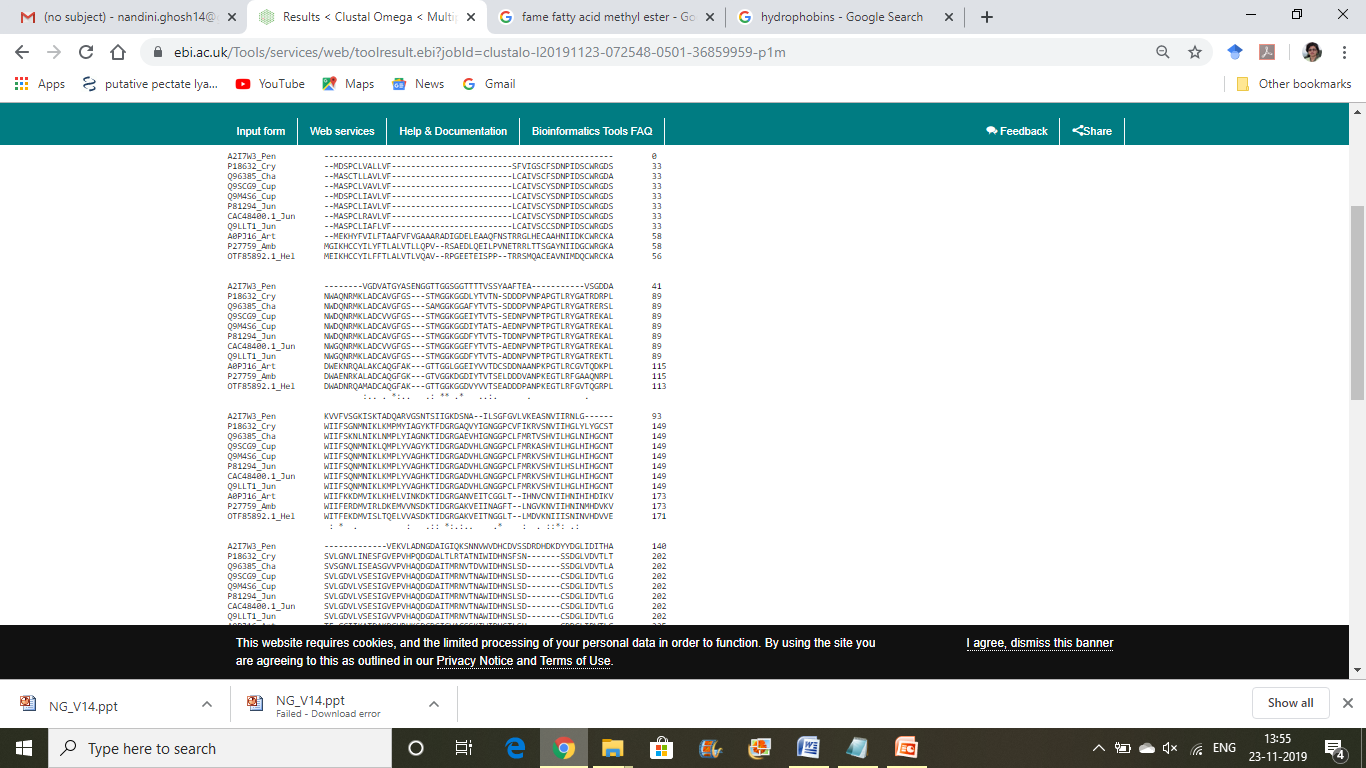

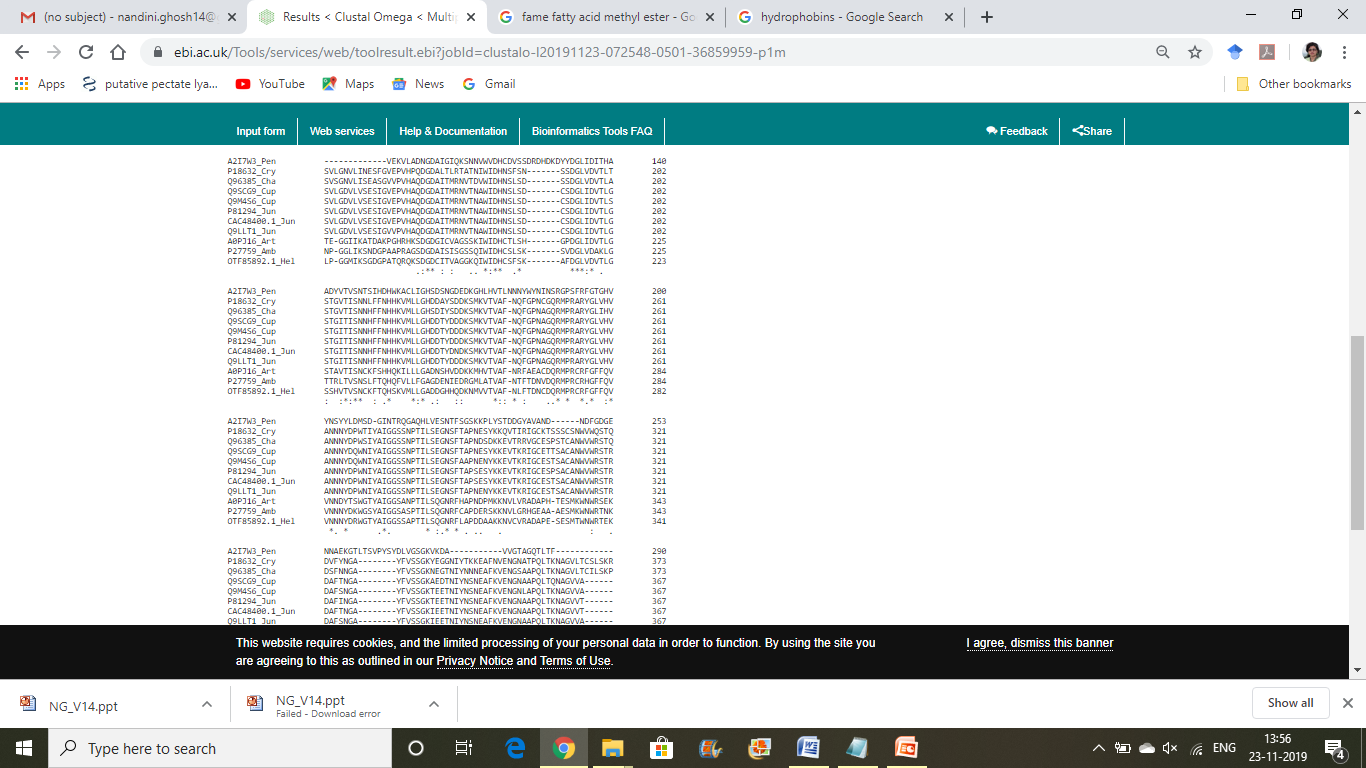

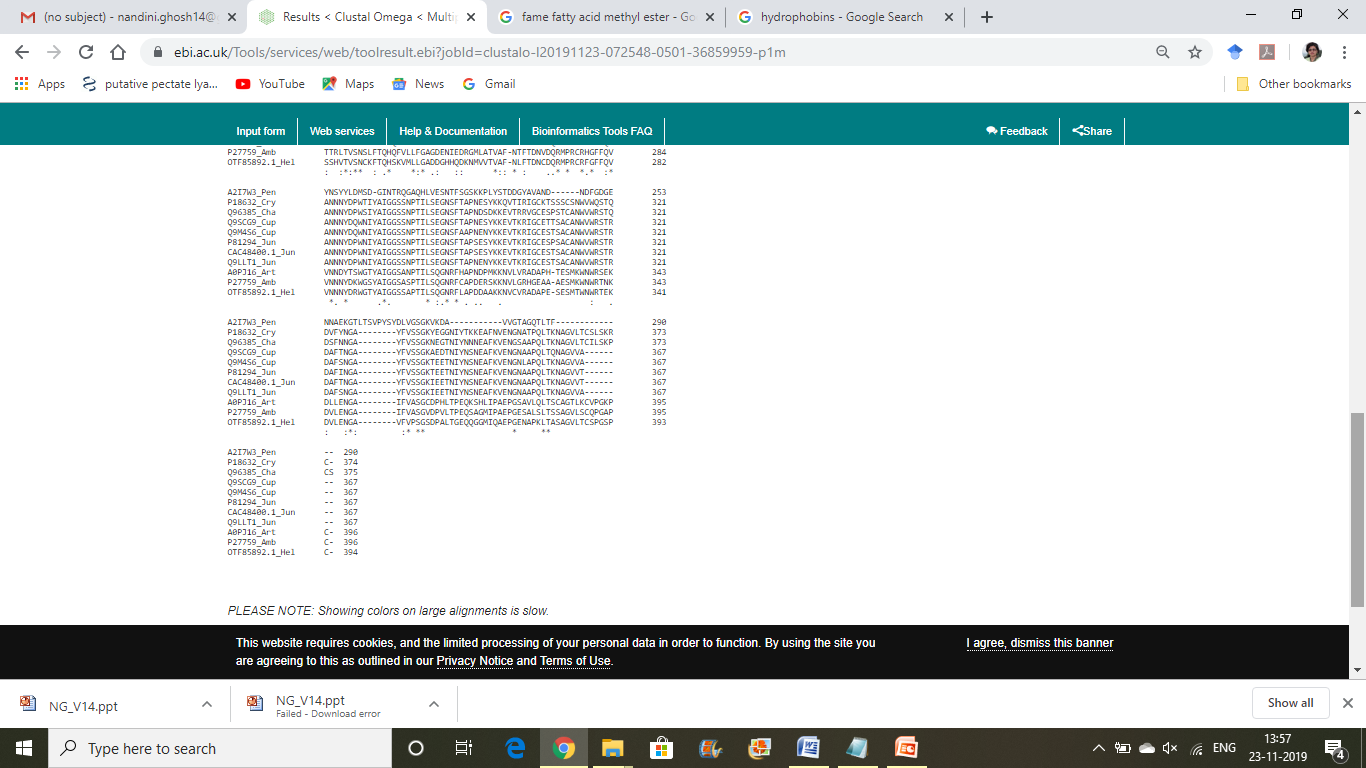
**

**Figure S1: Multiple sequence alignment of all the 10 pectate lyase allergens reported in IUIS database along with Hel a 6.**
